# Genetic determinants of global developmental delay and intellectual disability in Ukrainian children

**DOI:** 10.1186/s11689-024-09528-x

**Published:** 2024-03-27

**Authors:** Khrystyna Shchubelka, Liudmyla Turova, Walter Wolfsberger, Kelly Kalanquin, Krista Williston, Oleksii Kurutsa, Anastasiia Makovetska, Yaroslava Hasynets, Violeta Mirutenko, Mykhailo Vakerych, Taras K Oleksyk

**Affiliations:** 1 Department of Biological Sciences, Oakland University, 118 Library Drive, Rochester, Yaroslava Hasynets, MI 48309 USA; 2 Department of Biology, State University “Uzhhorod National University”, Voloshyna street, 32, Uzhhorod, 88000 Ukraine; 3 Department of Clinical Immunology and Allergology with Medical Genetics Section, National Bogomolets Medical University, Tarasa Shevchenko Blvd, 13, Kyiv, 01601 Ukraine; 4 118 Library Drive, Rochester, Yaroslava Hasynets, MI 48309 USA; 5 Faculty of Information Technologies, State University “Uzhhorod National University”, Zankovetska street, 89, Uzhhorod, 88000 Ukraine; 6 Medical Faculty #1, National Bogomolets Medical University, Taras Shevchenko Blvd, 13, Kyiv, 01601 Ukraine

**Keywords:** Global developmental delay, Intellectual disability, Next generation sequencing, Gene panel testing, Whole exome sequencing, Ukraine.

## Abstract

**Background:**

Global developmental delay or intellectual disability usually accompanies various genetic disorders as a part of the syndrome, which may include seizures, autism spectrum disorder and multiple congenital abnormalities. Next-generation sequencing (NGS) techniques have improved the identification of pathogenic variants and genes related to developmental delay. This study aimed to evaluate the yield of whole exome sequencing (WES) and neurodevelopmental disorder gene panel sequencing in a pediatric cohort from Ukraine. Additionally, the study computationally predicted the effect of variants of uncertain significance (VUS) based on recently published genetic data from the country’s healthy population.

**Methods:**

The study retrospectively analyzed WES or gene panel sequencing findings of 417 children with global developmental delay, intellectual disability, and/or other symptoms. Variants of uncertain significance were annotated using CADD-Phred and SIFT prediction scores, and their frequency in the healthy population of Ukraine was estimated.

**Results:**

A definitive molecular diagnosis was established in 66 (15.8%) of the individuals. WES diagnosed 22 out of 37 cases (59.4%), while the neurodevelopmental gene panel identified 44 definitive diagnoses among the 380 tested patients (12.1%). Non-diagnostic findings (VUS and carrier) were reported in 350 (83.2%) individuals. The most frequently diagnosed conditions were developmental and epileptic encephalopathies associated with severe epilepsy and GDD/ID (associated genes *ARX, CDKL5, STXBP1, KCNQ2, SCN2A, KCNT1, KCNA2*). Additionally, we annotated 221 VUS classified as potentially damaging, AD or X-linked, potentially increasing the diagnostic yield by 30%, but 18 of these variants were present in the healthy population of Ukraine.

**Conclusions:**

This is the first comprehensive study on genetic causes of GDD/ID conducted in Ukraine. This study provides the first comprehensive investigation of the genetic causes of GDD/ID in Ukraine. It presents a substantial dataset of diagnosed genetic conditions associated with GDD/ID. The results support the utilization of NGS gene panels and WES as first-line diagnostic tools for GDD/ID cases, particularly in resource-limited settings. A comprehensive approach to resolving VUS, including computational effect prediction, population frequency analysis, and phenotype assessment, can aid in further reclassification of deleterious VUS and guide further testing in families.

**Supplementary Information:**

The online version contains supplementary material available at 10.1186/s11689-024-09528-x.

## Background

Global developmental delay (GDD) and intellectual disability (ID) are terms used to describe individuals with significant delays in various developmental domains, including gross and fine motor skills, language and communication, and personal and social conduct [1]. While the designation GDD is reserved to children under the age of five, ID is applied for older children and adults. Both conditions are diagnosed when the standardized neurological tests fall two standard deviations below the age-appropriate mean [2]. While 40% of all GDD/ID cases are attributable to genetic disorders, other factors such as perinatal trauma, intrauterine infections, and toxic exposure, as well as postnatal events can also contribute to the developmental delay [3]. GDD/ID can also be accompanied by autism spectrum disorder (ASD) as well as anatomical abnormalities of other organ systems.

Traditionally, chromosomal microarray (CMA) and fragile X syndrome testing have been the primary diagnostic approaches for GDD/ID. However, CMA can only detect chromosomal deletions or duplications in about 20% of genetic cases. The advent of next-generation sequencing (NGS), specifically gene panel testing and whole exome sequencing (WES), has revolutionized the search for causative variants in neurodevelopmental disorders, increasing diagnostic success by an additional 25%. NGS techniques, with improved testing methods and bioinformatic algorithms, can now detect large copy number variations (CNVs) and chromosomal aberrations previously undetectable by NGS alone. Even though CMA and WES are not interchangeable, with the current improvements in testing techniques and bioinformatic algorithms, NGS gene panels and WES can accurately find large CNVs and chromosomal aberrations previously doomed undetectable by NGS technique [4].

Syndromic genetic disorders are the leading cause of pediatric disability in Ukraine [5]. However, the diagnosis of developmental delay in Ukraine has been delayed due to limited newborn screening and only the recent adoption of NGS genetic testing by physicians [6, 7].

Apart of isolated case reports, there has not been a comprehensive study on genetic causes of GDD/ID conducted in Ukraine, in particular, on diagnostic yield of NGS panels and WES techniques. This report will help pave the way to detecting locally significant candidate pathogenic variants for future variant resolution and familial studies in Ukraine and across Eastern Europe [8].

## Methods

This is a retrospective study of the cohort consisted of a mixed set of individuals diagnosed with GDD/ID only, as well as GDD/ID patients with ASD and/or multiple congenital anomalies or other functional symptoms. The diagnostic protocol was performed according to the Diagnostic and Statistical Manual of Mental Disorders (DSM-5, APA 2013) [9]. The patients were referred to a medical geneticist by either pediatrician or pediatric neurologist for consultation. All 416 children enrolled in the study underwent sequencing of whole exome sequencing (WES, Invitae Inc., San Francisco, CA) or custom broad neurodevelopmental disorder (NDD) gene panel sequencing (Invitae Inc., San Francisco, CA). The medical geneticist obtained consent for testing and signed standardized requisition forms with optional clinical and demographic information. Family follow-up has not yet been performed with these patients’ family members. The study of deidentified aggregated data was approved as “No Human Subject Research” by the Institutional Review Board of Oakland University (Rochester, MI, Study #RB-FY2023-120).

### NGS neurodevelopmental disorders panel and whole exome sequencing

The neurodevelopmental disorders panel (NDD) included 1,813 genes (Supplementary Table 1, sequencing and report limitations specified in the Supplementary file 1). For the NDD panel, genomic DNA from the submitted samples was extracted and enriched for targeted regions using the hybridization-based protocol [10]. For the WES sequencing, DNA libraries were prepared using the PCR-free method. The WES panel included a panel of more than 18,000 genes (Invitae Inc., San Francisco, CA).

All blood and saliva samples underwent double-step verification by visual identifiers (ID, Sex) and sex determined by sequencing, according to the company protocol (Invitae Inc., San Francisco, CA). All targeted regions were sequenced on Illumina NovaSeq 6000 platform (Illumina, San Diego, CA, USA). The average coverage for NDD panel testing was 50x, while for WES it was 35X across the entire exome.

Read mapping was performed to the reference GRCh37 human genome. To categorize the variants according to the laboratory, several pieces of evidence were considered, such as variant frequency and type, clinical findings, experimental research, and indirect and computational approaches.

Before being reported, clinically important variation that failed to meet strict NGS quality parameters had its accuracy verified by alternative methods [4], Sherloc [11], a points-based framework based on the joint consensus recommendations from the American College of Medical Genetics and Genomics and the Association for Molecular Pathology [12], was used to analyze the variations discovered by the bioinformatics pipeline. According to Invitae protocols, CNVs were confirmed through the application of either MLPA or Droplet Digital PCR (ddPCR). In cases where MLPA or ddPCR is unavailable, aCGH, was employed which involves a custom-designed microarray focused on exons.

Genomic data analysis.

Initially, a dataset was created in Ukraine containing reported genomic variants for each individual. This dataset also included information such as sex, age, and phenotypical descriptions. Subsequently, the data were transmitted to Oakland University, MI (USA). Due to the heterogeneous nature of data provided in the reports, the alleles with missing explicit rs-code were cross-referenced by their respective allele and protein notation according to Sequence Variant Nomenclature specifications, using the ENSEMBL Variant Recoder [13], resulting in the genomic positions of each reported variant for GRCh38 genomic reference and their relevant rs-codes. After the validation, we performed a detailed search on reported pathogenic (P) or likely pathogenic (LP) variants using ClinVar [14] and OMIM [15] databases.

Variants of uncertain significance (VUS) or heterozygous variants related to the autosomal recessive condition were considered non-diagnostic. Using genome data from 97 individuals from the “Genome Diversity in Ukraine” database [16, 17], as well as from the 150 whole genomes from the database of the cross-border cooperation project “Partnership for Genomic research in Ukraine and Romania” [18], we performed an additional annotation for the effect prediction among the VUS using CADD and SIFT scores and estimated their frequency in the general population of Ukraine.

## Results

### Demographic and clinical characteristics of the cohort

The cohort included 416 exclusively pediatric patients under 18 years old (age ranged between 1 and 18 years), with 60.9% males. Both sexes, males and females, had a similar mean age of around 7 yo (Table 1). Genetic information either from the NDD gene panel or from WES results as a first- or second-line test after inconclusive CMA was available for analysis in all individuals in this study. Diagnostic data for karyotypes, chromosomal microarrays, or FMR1 CGG-repeat expansion tests for the Fragile X syndrome, was not included in this study. Demographic and clinical information included in the analysis is summarized in Table 1.

**Table 1:** Demographic and clinical characteristics of the studied cohort

Characteristic	Cohort (*N* = 416)
**Sex**	
Males N(%)	253 (60.9)
Female N(%)	163 (39.1)
**Years of age**	
Males Mean (SD)	7.41 (4.04)
Females Mean (SD)	7.37 (3.86)
**Comorbidities**	
ASD N (%)	230 (55.1)
Confirmed Congenital Anomalies/Additional symptoms N (%)	69 (16.5)
Epilepsy N (%)	121 (29.08)
**Genetic Testing**	
Neurodevelopmental Disorders Panel N (%)	379 (91.1)
WES N (%)	37 (8.8)

### Yield of definitive the molecular diagnosis

**Fig. 1:**
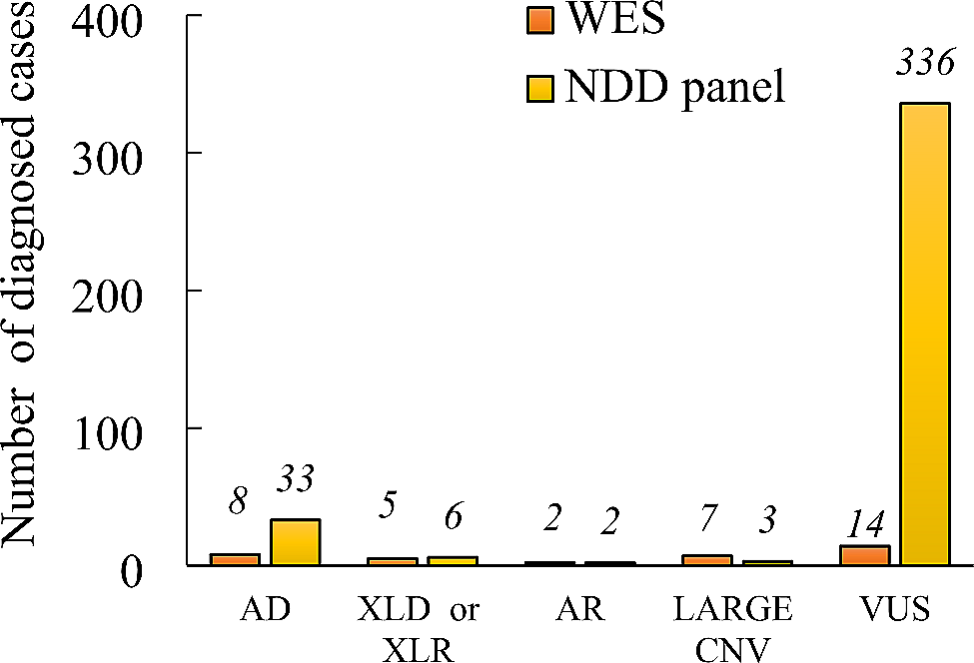
The absolute number diagnosed conditions by mode of inheritance: autosomal dominant (AD), autosomal recessive (AR), X-linked dominant (XLD), and X-linked recessive (XLR) (Table 2) diagnosed by either WES or NDD panel. Large CNVs are shown separately (Table 3)

We identified a definitive molecular diagnosis in 66 or 16.3% of all individuals (Fig. 1). In general, WES positively diagnosed 22 out of 37 ordered cases (59.4%), while the NGS testing panel yielded 44 definitive diagnoses among the 379 tested patients (12.1%). Non-diagnostic variants (VUS and carrier) were identified in 348 (83.4%) individuals (details in Fig. 1).

Most of the known diagnosed conditions followed the AD mode of inheritance (41, or 62.11%), four with AR, nine with XLD, and two with XLR modes (Table 2). Compound heterozygosity was confirmed by segregation analysis. Chromosome 15 was most affected by these types of variants. The most commonly diagnosed same-gene condition was Rett syndrome: five cases were caused by single nucleotide variants (SNVs) or small indels in the *MECP2* gene. Among the other diagnoses, 12 different conditions were observed in two individuals each, all the rest diseases were isolated cases only (Table 2). Out of 66 diagnosed cases, the rest 10 patients harbored large copy number variations (CNVs) encompassing multiple genes (15.1% of diagnosed cases) (Table 3).

**Table 2:** Summary of diagnosed conditions and causative variants in the cohort, classified by the mode of inheritance (see Fig 0.1)

N	Patient ID	Gene	Codon and amino acid change	Effect on protein	Position on chromosome GRCh38.p13	Chromosome	Number of diagnosed cases	OMIM ID	Disorder
**Autosomal dominant mode of inheritance (N = 41)**									
*1*	*190*	*ATP1A3*	c.2851G > A (p. Glu951Lys)	missense	41,967,732	19q13.2	1	614,820	Alternating hemiplegia of childhood 2
*2*	*55*	*C19ORF12*	c.204_214del (p. Gly69Argfs*10)	frameshift	29,702,957–29,702,977	19q12	1	614,298	Neurodegeneration with brain iron accumulation 4
*3*	*147*	*CHD1*	c.206 C > G (p.Ser69Pro)	missense	98,904,946	5q15-q21.1	1	617,682	Pilarowski-Bjornsson syndrome
4	139	CTBP1	c.991 C > T(p.Arg331Trp)	missense	1,213,028	4p16.3	1	617,915	Hypotonia, ataxia, developmental delay, and tooth enamel defect syndrome
*5*	*278*	*DEPDC5*	c.1325-1G > A	splice acceptor	31,810,520	22q12.2-q12.3	1	604,364	Epilepsy, familial focal, with variable foci 1
*6*	*14*	*DYRK1A*	c.1294G > T	stop gained	37,505,364	21q22.13	1	614,104	Intellectual developmental disorder, autosomal dominant 7
*7*	*101*	*FOXG1*	c.701 C > T (p.Ser234Phe)	missense	28,767,980	14q12	2	613,454	Rett syndrome, congenital variant, FOXG1 syndrome
*8*	*392*		c.587 A > C (p.Gln196Pro)	missense	28,767,866				
*9*	*108*	*GABRB3*	c.288G > T (p.Arg96Ser)	missense	26,621,487	15q12	2	617,113	Developmental and epileptic encephalopathy 43
*10*	*23*		c.905 A > G (p.Tyr302Cys)	missense	26,561,107				
*11*	*314*	*GLRA1*	c.381dup (p.Phe128Leufs*11)	frame shirt	15,185,987	5q33.1	1	149,400	Hyperekplexia 1
*12*	*161*	*GNB1*	c.239T > C (p.Ile80Thr)	missense	1,806,503	1p36.33	1	616,973	Intellectual developmental disorder, autosomal dominant 42
*13*	*151*	*KCNA2*	c.997T > C (p.Phe333Leu)	missense	110,603,786	1p13.3	2	616,366	Developmental and epileptic encephalopathy 32
*14*	*376*		c.1219 C > T (p.Pro407Ser)	missense	110,603,564				
*15*	*65*	*KCNQ2*	c.1639 C > T (p.Arg547Trp)	missense	63,413,574	20q13.33	1	613,720	Developmental and epileptic encephalopathy 7
*16*	*237*	*KCNT1*	c.1309 C > T (p.Leu437Phe)	missense	135,765,732	9q34.3	2	614,959	Developmental and epileptic encephalopathy 14
*17*	*323*		c.784 C > T (p.Arg262Trp)	missense	135,758,438				
*18*	*131*	*KDM1A*	c.2410dupA (p.Ser785Leufs*22)	frame shift	23,082,331	1p36.12	1	616,728	Cleft palate, psychomotor retardation, and distinctive facial features
*19*	*68*	*KMT2C*	c.8965_8970delinsAGTACCTT (p.Val2989Serfs*44)	missense	118,504,857	7q36.1	1	617,768	Kleefstra syndrome 2
*20*	*290*	*KMT2D*	c.14,710 C > T (p.Arg4904*)	stop gained	49,027,256	12q13.13	1	147,920	Kabuki syndrome 1
*21*	*134*	*MACF1*	c.7661 A > G (p.Gln2554Arg)	missense	39,382,151	1p34.3	1	618,325	Lissencephaly 9 with complex brainstem malformation
*22*	*44*	*KMT2A*	c.2968_2969insAGAG (p.Cys990*)	nonsense	118,474,126	11q23.3	2	605,130	Wiedemann-Steiner syndrome
*23*	*72*		c.1038del (p.Val347fs)	frame shift	118,472,196				
*24*	*172*	*NPRL3*	Exon 2–6 deletion	truncating	112,622 - 138,334	16p13.3	1	617,118	Epilepsy, familial focal, with variable foci 3
*25*	*182*	*PAFAH1B1*	c.1159 + 2T > A	splice donor	2,680,322	17p13.3	2	607,432	Lissencephaly 1
*26*	*284*		c.656G > A (p.Trp219*) (mosaic)	nonsense	2,674,239				
*27*	*150*	*PTPN11*	c.922 A > G (p.Asn308Asp)	missense	112,477,719	12q24.13	1	163,950	Noonan syndrome 1
*28*	*389*	*SCN1A*	c.4073G > T (p.Trp1358Leu)	missense	166,002,683	2q24.3	2	607,208	Dravet syndrome
*29*	*403*		c.4265 A > G (p.Tyr1422Cys)	missense	166,002,491				
*30*	*306*	*SCN2A*	c.2552 C > A (p.Ser851*)	nonsense	65,342,459	2q24.3	2	613,721	Developmental and epileptic encephalopathy 11
*31*	*283*		Exon 17 deletion						
*32*	*311*	KCNC1	c.22G > T(p. .Glu8Ter)	missense	17,736,024	11p15.1	1	616,187	Epilepsy, progressive myoclonic 7
*33*	*130*	*STXBP1*	c.1606delC		127,682,459	9q34.11	2	612,164	Developmental and epileptic encephalopathy 4
*34*	*224*		c.175G > A (p.Glu59Lys)	missense	127,658,380				
*35*	*257*	*SYNGAP1*	c.1564del (p.Glu522Lysfs*5)	frame shirt	33,438,273	6p21.32	2	612,621	Developmental disorder, autosomal Intellectual dominant 5
*36*	*126*		c.1534G > T (p.Glu512Ter)	stop gained	33,438,777				
*37*	*142*	*TGFBR1*	c.844T > C (p.Tyr282His)	missense	99,142,574	9q22.33	1	609,192	Loeys-Dietz syndrome 1
*38*	*116*	*TLK2*	c.754 C > T (p.Gln252Ter)	stop gained	62,560,049	17q23.2	1	618,050	Intellectual developmental disorder, autosomal dominant 57
*39*	*186*	*TREX1*	c.341G > A (p.Arg114His)	missense	48,466,996	3p21.31	2	225,750	Aicardi-Goutieres syndrome 1
*40*	*378*		c.341G > A (p.Arg114His)	missense	48,466,996				
*41*	*145*	*TRRAP*	c.6653 A > C (p.Glu823Ala)	missense	98,967,697	7q22.11	1	618,454	Developmental delay with or without dysmorphic facies and autism
***Autosomal recessive mode of inheritance*** (*N* = 4)									
42	16	ARSA	c.465 + 1G > A	splice donor	50,627,165	22q13.33	1	250,100	Metachromatic leukodystrophy
			c.542T > G (p.Ile181Ser)	stop gained	50,626,976				
43	180	FBXL4	c.45T > G (p.Tyr15*) c.627_633del (p.Asn210Leufs*9)	frame shift	98,926,944	6q16.1-16.2	1	615,471	Mitochondrial DNA depletion syndrome 13 (encephalomyopathic type)
44	56	NPC1	c.2861 C > T (p.Ser954Leu)	missense	23,539,405	18q11.2	1	257,220	Niemann-Pick disease, type C1
			c.1026G > A (p.Trp342*)	nonsense	23,556,543				
45	332	PAH	c.1222 C > T (p.Arg408Trp)	missense	102,840,493	12q23.2	1	261,600	Phenylketonuria
			c.473G > A (p.Arg158Gln)	missense	102,866,632				
***X-linked dominant*** (*N* = 9)									
*46*	*42*	*CDKL5*	c.372_385del (p.His124Glnfs*2)	frame shift	18,579,937	Xp22.13	1	300,672	Developmental and epileptic encephalopathy 2
*47*	*251*	*MECP2*	c.397 C > T (p.Arg133Cys)	missense	154,031,431	Xq28	5	312,750	Rett syndrome
*48*	*83*		c.806del (p.Gly269Alafs*20)	frame shift	154,031,025				
*49*	*414*		c.1084_1216del (p.Pro362Argfs*3)	frame shift	154,030,743				
*50*	*106*		c.844del (p.Arg282fs)	frame shift	154,031,020				
*51*	*95*		c.1115 C > A (p.Ser372Ter)	stop gained	154,030,749				
*52*	*157*	*SLC35A2*	c.845G > A (p.Gly221Glu)	missense	48,905,064	Xp11.23	1	300,896	Congenital disorder of glycosylation, type IIm
*53*	*60*	*WDR45*	c.1013_1014del (p.Phe338Tyrfs*3)	frame shift	49,074,874	Xp11.23	2	300,894	Neurodegeneration with brain iron accumulation 5
*54*	*61*		c.64del (p.Cys22Alafs*16)	frame shift	49,077,902				
***X-linked recessive*** (*N* = 2)									
*55*	*400*	*ATP7A*	c.2938 C > T (p.Arg980Ter)	stop gained	78,029,271	Xq21.1	1	309,400	Menkes disease
*56*	*200*	*ARX*	c.1058 C > T (p.Pro353Leu)	missense	25,012,937	Xp21.3	1	308,350	Developmental and epileptic encephalopathy 1

**Table 3:** Diagnostic large CNV spanning multiple genes

Case N	Patient ID	Mutation	Genes duplicated/deleted	# of allele copies	Condition	OMIM or Orphanet ID	ACMG class
**1**	276	2q37 deletion	AGXT; D2HGDH; KIF1A; NDUFA10	1	Chromosome 2q37 deletion syndrome	600,430	PVS1
***2***	352	2p16.3 deletion	*NRXN1*, Exons 2–3	1	2p16.3 deletion syndrome	614,332	PS1-4
***3***	51	4.16 duplication	*CC2D2A; CPLX1; CTBP1; EVC; EVC2; FGFRL1; IDUA; KLB; LETM1; PIGG; QDPR; RBPJ; SEPSECS; WHSC1*	3	4.16 microduplication syndrome	ORPHA:96,072	PVS1
***4***	84	5p13 duplication	*SLC6A19; AMACR; HCN1; NADK2; NDUFS6; SLC6A3*	mosaic	Chromosome 5p13 duplication syndrome	613,174	PVS1
***5***	328	15q24 deletion	*CYP11A, SIN3A*	1	15q24 deletion syndrome, Witteveen-Kolk syndrome	613,406	PVS1
***6***	189	15q11.2 deletion	*UBE3A; GABRB3*	1	Angelman syndrome	615,656	PVS1
***7***	398	15q11.2 duplication	*UBE3A; GABRB3*	3	Chromosome 15q11-q13 duplication syndrome	608,636	PVS1
***8***	371	17p11.2 deletion	*ALDH3A2; TOP3A; ATPAF2*	1	17p11.2 deletion syndrome	182,290	PVS1
***9***	353	20P duplication	*ATRN; ITPA; NDUFAF5; PANK2; PDYN; PLCB1; PRNP; SNRPB; TBC1D20*	3	Trisomy 20p	ORPHA:261,318	PVS1
***10***	66	X28 duplication	*FLNA; NAA10; MECP2*	2	Intellectual developmental disorder, X-linked syndromic, Lubs type	300,260	PVS1

The prevailing group of diseases diagnosed were classified as developmental and epileptic encephalopathies (type 1, 2, 4, 7, 11, 14, 32 and 43) characterized by severe epilepsy and GDD/ID (14 cases). The specific variants reported for each definitive diagnosis are reported in the Table 2.

### Annotation and analysis of variants of uncertain significance

**Fig. 2:**
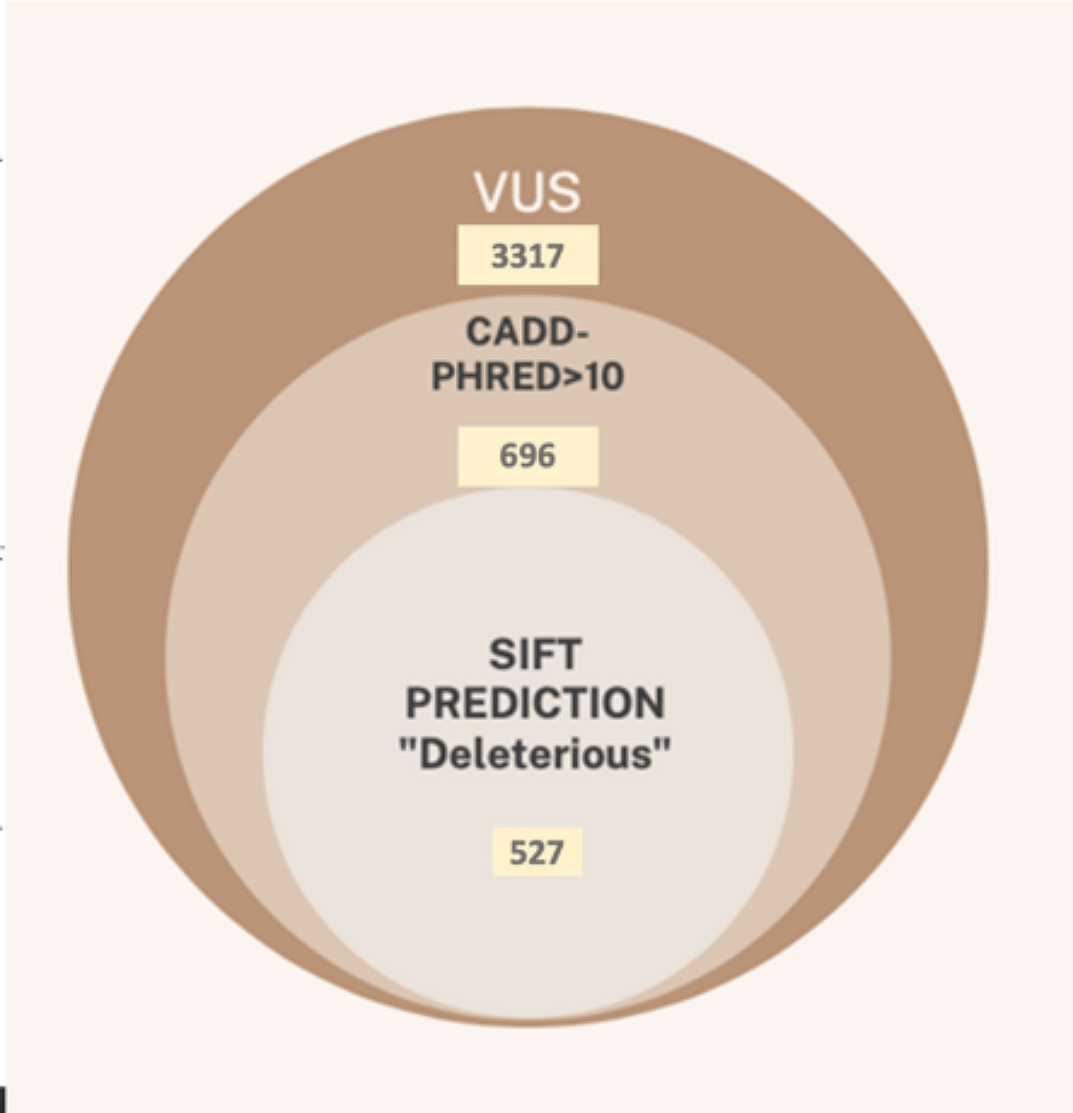
The Venn diagram showing an overlap in distributions of alleles with high CADD-Phred score [19] and “deleterious” SIFT-prediction score [20]

A total of 3,317 heterozygous variants of uncertain significance (or VUS) were identified in our cohort of 417 patients. In this VUS dataset, a CADD-Phred score between 10 and 20 were associated with 245 variants (considered 10% most deleterious substitutions in the human genome), while for 723 variants it was above 20 (the top 1% most deleterious variants) [19]. A deleterious SIFT-prediction score [20] for least one alternative transcript was calculated for 527 variants (Fig. 2; see details in Supplementary Table 2).

Among these deleterious variants identified in the study,165 were in the genes associated with AD conditions and 56 associated with X-linked dominant or recessive conditions according to OMIM (a total of 221). Here we reported these variants as potentially diagnostic and suggested a segregation analysys in 138 undiagnosed cases (Supplementary Table 2). However, 18 of these 221 variants had allele count 1 or 2 in 247 among the unaffected individuals from Ukraine (Supplementary Table 2). The rest of the variants (203) were absent in healthy individuals. Gene *PACS2* (associated with AD developmental and epileptic encephalopathy 66, OMIM 618,067) was most frequently altered (seven individuals harboring rare highly deleterious heterozygous SNV) (Supplementary Table 2).

## Discussion

Global developmental delay and intellectual disability (GDD/ID) is usually diagnosed for the patients with developmental delay before the exact genetic diagnosis is established [21].

GDD/ID is a complex set of symptoms with a wide range of genetic causes, including single nucleotide variants, large chromosomal indels, and copy number variants. However, in Ukraine a comprehensive study on the genetic causes of GDD/ID has not been conducted yet. This is mainly due to only the recent availability of NGS-based diagnostic tests. Also, the whole-genome data on the general genetic composition of the population has just been published recently [8, 16, 17], there was a need to use genome data available to evaluate the diagnostic yield of WES and NGS gene panel.

In this study, we report the largest to-date descriptive dataset of diagnosed genetic conditions which present with GDD/ID as a part of the clinical picture. Also, we report a combined diagnostic yield of the NDD gene panel of 1813 genes and WES at 16.3% on previously undiagnosed cases. Expectedly, individually WES had a much higher diagnostic yield compared to the NDD gene panel (59.4% and 12.1% respectively). Similarly, the reported diagnostic yield of target exome sequencing in patients with ID ranges from 21 to 55.7%. Pekeles et al. [22]. used four distinct panels in a trial with a sample of 48 patients and achieved a 21% rate of definitive diagnoses. With a sample of 133 patients, Yamamoto et al. [23] achieved a diagnostic rate of 29.3%. A similar rate of 34% was reported by Gieldon and colleagues in a study using 4 813 gene panels in 106 patients [24]. It is possible that the significant difference in genetic yield between individuals who underwent exome sequencing and those who received targeted sequencing could be attributed to the bias due limited sample size of 37 WES administered might have led to some variability in the results. In such a small sample, the observed difference could have occurred randomly without any underlying phenotypic differences between the groups. Also, the NDD panels were prescribed in many cases due to their significantly lower cost. However, it is also important to consider other factors that could contribute to the observed discrepancy. Exome sequencing is a more comprehensive approach compared to targeted sequencing, as it examines a larger portion of the genome. This broader coverage increases the likelihood of identifying disease-causing genetic variants, leading to a higher genetic yield. Additionally, exome sequencing may capture variants in genes that are not initially suspected based on the clinical presentation but still contribute to the observed phenotype.

Our study showed that both the NGS gene panel and WES can be diagnostic of large CNVs associated with the clinical picture of known syndromes in the absence of CMA testing. In these 10 cases, both the NDD gene panel and WES reported multiple whole genes deleted or multiplied, which was indicative of the cytogenic location to determine large aberration from the set of genes mutated.

Most of the reported variants in our cohort were variants of uncertain significance (VUS). Both patients and medical geneticists face challenges as a result of the discovery of a VUS [25]. A VUS may ultimately be reclassified as pathogenic or benign, but this process often takes several years and may never be completed for rare VUS, particularly if the condition is uncommon to find enough cases or too expensive to test relatives [26]. The clinical relevance of a VUS has been increasingly determined by a phenotypically driven in-silico approach [27]. Furthermore, variant interpretation can be enhanced by quantitative analysis of consortium disease cohorts and population controls [28].

The absence of family members’ genetic data was a major limiting factor to fully classifying or resolving the effect of the variants of uncertain significance in our study. Also, this prevented us from identifying de novo variants. Thus, we performed their annotation using CADD and SIFT predictive scores and found that as many as 527 variants were classified as deleterious by both scores (CADD-Phred > 10 and SIFT prediction “Deleterious”) with MAF < 0.01 and should be resolved for disease causation by family testing or phenotype confirmation tests. Out of these, 221 variants were associated with AD or X-linked conditions making them potentially diagnostic. This number of variants resolved could potentially increase the diagnostic yield in 138 undiagnosed case (by 33%). Interestingly, having WGS and phenotype data of 249 Ukrainians, we found that 18 of 221 potentially diagnostic variants are present at very low frequency in unaffected individuals (Supplementary Table 2), implying they might not be disease causative even with high prediction scores. Other 203 variants were absent in the sample of healthy individuals. Importantly, some of the VUS with high prediction scores and associated with AD or X-linked conditions were found in diagnosed individuals, potentially making their condition associated with multiple genetic aberrations.

## Conclusions

This is the first comprehensive study on genetic causes of GDD/ID conducted in Ukraine, including diagnostic yield of NDD gene panel and WES techniques, comparing data from the cohort of pediatric patients and general population in the country. We report largest to date descriptive dataset of diagnosed genetic conditions which present with GDD/ID as a part of the clinical picture. Our results support the important role of NGS gene panel and WES in the diagnostic approach to GDD/ID cases as a first line choice in the scenario of scarce financial resources and logistic difficulty to perform multiple genetic tests in a family. A comprehensive approach to VUS resolution including computational effect prediction, comparative analysis of allele frequencies in population controls and phenotype assessment can be of extreme help to fully classify deleterious variants and narrow down the list for the further family follow-up testing.

### Electronic supplementary material

Below is the link to the electronic supplementary material.

Supplementary Material 1

Supplementary Material 2

Supplementary Material 3

Supplementary Material 4

## Data Availability

The data are available from the corresponding author on reasonable request.

## Abbreviations

GDD: Global Developmental Delay
ID: Intellectual Disability
WES: Whole Exome Sequencing
NGS: Next Generation Sequencing
AD: Autosomal Dominant
AR: Autosomal Recessive
XLD: X-Linked Dominant
XLR: X-Linked Recessive
CNVs: Copy Number Variants
NDD: Neurodevelopmental Disorders
