# Supplementary material for: Genetic determinants of global developmental delay and intellectual disability in Ukrainian children
Source: J Neurodev Disord. 2024 Mar 27;16:13. doi: 10.1186/s11689-024-09528-x (PMC10967201; doi:10.1186/s11689-024-09528-x)
Supplement: Supplementary file 3 — Supplementary Material 3 [file 11689_2024_9528_MOESM3_ESM.docx]

**Limitations of the NDD panel**

**Sited from the generic report of the broad NDD panel**

Based on validation study results, this assay achieves >99% analytical sensitivity and specificity for single nucleotide variants, insertions and deletions <15bp in length, and exon-level deletions and duplications. Invitae's methods also detect insertions and deletions larger than 15bp but smaller than a full exon but sensitivity for these may be marginally reduced. Invitae's deletion/duplication analysis determines copy number at a single exon resolution at virtually all targeted exons. However, in rare situations, single-exon copy number events may not be analyzed due to inherent sequence properties or isolated reduction in data quality. Certain types of variants, such as structural rearrangements (e.g. inversions, gene conversion events, translocations, etc.) or variants embedded in sequence with complex architecture (e.g. short tandem repeats or segmental duplications), may not be detected.

Additionally, it may not be possible to fully resolve certain details about variants, such as mosaicism, phasing, or mapping ambiguity. Unless explicitly guaranteed, sequence changes in the promoter, non-coding exons, and other non-coding regions are not covered by this assay. Please consult the test definition on our website for details regarding regions or types of variants that are covered or excluded for this test. This report reflects the analysis of an extracted genomic DNA sample. While this test is intended to reflect the analysis of extracted genomic DNA from a referred patient, in very rare cases the analyzed DNA may not represent that individual's constitutional genome, such as in the case of a circulating hematolymphoid neoplasm, bone marrow transplant, blood transfusion, chimerism, culture artifact or maternal cell contamination. Invitae's RNA analysis is not designed for use as a stand-alone diagnostic method and cannot determine absolute RNA levels. Results from the RNA analysis may not be informative for interpreting copy number gains.

*MTHFR:* The NM_005957.4: c.665C>T (p.Ala222Val) (aka 677C>T) and c.1286A>C (p.Glu429Ala) (aka 1298A>C) variants are not reported in our primary report.

*TTN:* Exons 45-46, 147, 149, 164, 172-201 (NM_001267550.2) are excluded from analysis. *TTN* variants are included in the primary report based on functional effect and/or location. A complete list of variants of uncertain significance, likely benign and benign variants in *TTN* available upon request. Variants are named relative to the NM_001267550.2 (meta) transcript. Variants in the coding sequence and intronic boundaries of the clinically relevant NM_133378.4 (N2A) and fetal isoforms are reported (PMID: 25589632, 29598826, 29691892, 31660661), with the exception of the *PEVK* tandem repeat region (172-198) (PMID: 28040389).

*SDHC:* Sequencing analysis for exons 2, 6 includes only cds +/- 10 bp.

*SLC9A6:* Sequencing analysis for exons 14 includes only cds +/- 10 bp.

*CTDP1:* Sequencing analysis is not offered for exon 8.

*AR:* CAG repeat numbers are not determined. *S*

*LC25A26*: Deletion/duplication analysis is not offered for exon 5.

*TBCE:* Sequencing analysis for exons 2 includes only cds +/- 10 bp.

*UBE3B*: Sequencing analysis for exons 6 includes only cds +/- 0 bp.

*ATP1A1:* Deletion/duplication analysis is not offered for exon 1.

*USP53:* Sequencing analysis for exons 15 includes only cds +/-10 bp.

*FLNC:* Deletion/duplication analysis is not offered for exon 47. Sensitivity and specificity for single nucleotide variants, insertions and deletions in exons 47-48 may be reduced due to the presence of segmental duplications overlapping the region.

*GFPT1*: Sequencing analysis for exons 20 includes only cds +/- 10 bp.

*MEGF8*: Deletion/duplication analysis is not offered for exon 39. Sequencing analysis for exons 39 includes only cds +/- 10 bp.

*RANBP2:* Deletion/duplication and sequencing analysis is not offered for exons 1-11, 15-29. *CDK13:* Sequencing analysis for exons 12 includes only cds +/- 10 bp.

*PDSS1:* Deletion/duplication analysis is not offered for exon 2.

*CACNA1G:* Sequencing analysis for exons 2 includes only cds +/-0 bp.

*DENND5A:* Sequencing analysis for exons 9 includes only cds +/- 10 bp.

*AMN:* Deletion/duplication analysis is not offered for exon 1. ATM: Sequencing analysis for exons 6, 24, 43 includes only cds +/- 10 bp.

*KANSL1:* Deletion/duplication and sequencing analysis is not offered for exons 2-3.

*RNF13:* Sequencing analysis is not offered for exon 4. SNX14: Deletion/duplication analysis is not offered for exon 9. *ACACA:* Sequencing analysis for exons 14 includes only cds +/- 10 bp. *RARS*: Deletion/duplication analysis is not offered for exon 14. Sequencing analysis for exons 14 includes only cds +/- 10 bp.

*NAA35:* Sequencing analysis for exons 3 includes only cds +/- 10 bp.

*COX10:* Deletion/duplication and sequencing analysis is not offered for exon 6.

*HHAT:* Sequencing analysis for exons 3 includes only cds +/- 10 bp.

*VPS13A:* Deletion/duplication analysis is not offered for exons 2-3, 27-28.

*FGF12*: Sequencing analysis for exons 4 includes only cds +/- 0 bp.

*SEC63:* Sequencing analysis for exons 4, 13 includes only cds +/-0 bp.

*ABCC2:* Deletion/duplication analysis is not offered for exons 24-25.

*NFIA:* Sequencing analysis for exons 9 includes only cds +/- 0 bp.

*SMARCA2:* Deletion/duplication analysis is not offered for exons 4-5 and sequencing analysis is not offered for exon 4.

*TUBB2A*: Deletion/duplication and sequencing analysis is not offered for exon 2.

*RNF216:* Deletion/duplication and sequencing analysis is not offered for exons 2, 6.

*AK2:* Deletion/duplication and sequencing analysis is not offered for exon 6.

*RERE:* Sequencing analysis is not offered for exon 19.

*EZH2:* Sequencing analysis for exons 20 includes only cds +/- 10 bp.

*CSTB:* Dodecamer repeat numbers in the 5' UTR are not determined. *SMN1* or *SMN2:* The *SMN1* gene is identical to the *SMN2* gene with the exception of exon 8 (typically referred to as exon 7). This assay unambiguously detects *SMN1* exon 8 copy number and sequence variants. Sequence variants outside of exon 8 will also be detected, but this assay cannot determine whether the variant is located in *SMN1* or *SMN2.* *SMN2* exon 8 copy number and the SMN2 exon 8 c.859G>C (p.Gly287Arg) modifier variant will be reported for individuals with a positive result in *SMN1.* CNVS of exons 1-7 of *SMN1* or *SMN2* (typically referred to as exons 1-6 in the literature) will not be reported. Variants in all exons with no evidence towards pathogenicity are not reported but are available upon request. This assay cannot detect silent carriers (individuals that have 2 functional copies of S*MN1* on one chromosome and zero copies on the other). Therefore, a negative result for carrier testing greatly reduces but does not eliminate the chance that person is a carrier. For individuals with 2 copies of *SMN1*, the residual risk of being a carrier has been reported to be 1 in 121 in African Americans, 1 in 345 in Ashkenazi Jewish individuals, 1 in 628 in Asians, 1 in 632 in Caucasians, and 1 in 1061 in Hispanic individuals (PMID: 23788250). The SMA-STAT test does not detect sequence variants in *SMN1* or *SMN2*, and therefore cannot be used to identify compound heterozygotes.

*PRODH:* Deletion/duplication analysis is not offered for exons 8, 12.

*NEB*: Deletion/duplication analysis is not offered for exons 82-105. *NEB* variants in this region with no evidence towards pathogenicity are not included in this report, but are available upon request. *N*

*OTCH2:* Deletion/duplication and sequencing analysis is not offered for exons 1-4.

*OAT:* Deletion/duplication analysis is not offered for exon 2.

*CACNA1A:* Trinucleotide repeat expansions are not determined on this assay.

*DDC:* Deletion/ duplication analysis is not offered for exons 10-11.

*SELENON:* Deletion/duplication analysis is not offered for exon 1. GALC: Deletion/duplication analysis is not offered for exon 6.

*PRNP:* Octapeptide repeat numbers are not determined.

*DNAJC6:* Sequencing analysis for exons includes only cds +/- 10 bp.

*NIPBL:* Sequencing analysis is not offered for exon 33. IDS: Detection of complex rearrangements not offered (PMID: 7633410, 20301451).

*PACS1*: Sequencing analysis is not offered for exon 1. BBS9: Deletion/duplication analysis is not offered for exon 4.

*CFTR:* Sequencing analysis for exons 7 includes only cds +/- 10 bp.

*COL3A1:* Deletion/duplication analysis is not offered for exons 23-24.

*CLCNKB:* Deletion/duplication analysis is not offered for this gene.

*SNORD118:* Analysis of the 3' downstream flanking region of the SNORD118 gene is not offered. *AKR1D1*: Sequencing analysis for exons 4 includes only cds +/- 0 bp.

*NF1:* Sequencing analysis for exons 2, 7, 25, 41, 48 includes only cds +/- 10 bp.

*PKHD1:* Deletion/duplication analysis is not offered for exon 13.

*PTEN:* Sequencing analysis for exons 8 includes only cds +/- 10 bp. GPC3: Sequencing analysis for exons 3 includes only cds +/- 10 bp.

*TSFM:* Sequencing analysis is not offered for exon 5.

*OCLN*: Deletion/duplication and sequencing analysis is not offered for exons 5-9.

*GALM*: Deletion/duplication analysis is not offered for exons 5-7.

*NDUFB11*: Deletion/duplication and sequencing analysis is not offered for exon 1.

*PSPH:* Deletion/duplication and sequencing analysis is not offered for exons 4-5.

*PCLO:* Sequencing analysis for exons 2 includes only cds +/- 0 bp.

*CACNA1B*: Deletion/ duplication and sequencing analysis is not offered for exons 1-3.

*COL4A2:* Deletion/duplication and sequencing analysis is not offered for exon 21.

*PHIP*: Deletion/duplication analysis is not offered for exons 3-4.

*KANK1*: Sequencing analysis for exons 9 includes only cds +/-0 bp.

*ARX:* Analysis is validated to detect polyalanine expansions, but sensitivity may be reduced. *CACNA1C:* Deletion/duplication and sequencing analysis is not offered for exons 44-45.

*FH:* Sequencing analysis for exons 9 includes only cds +/- 10 bp.

*TPM3:* Deletion/duplication analysis is not offered for exon 10.

*FASN:* Sequencing analysis for exons 7 includes only cds +/- 10 bp.

*PAPSS2:* Sequencing analysis is not offered for exon 3. Sequencing analysis for exons 10 includes only cds +/- 10 bp.

*TUBG1:* Deletion/duplication and sequencing analysis is not offered for exon 9.

*BPTF:* Deletion/duplication and sequencing analysis is not offered for exon 8.

*TBX19:* Sequencing analysis for exons 3 includes only cds +/- 5 bp.

*NARS2:* Sequencing analysis for exons 12 includes only cds +/- 0 bp.

*PIGB:* Deletion/duplication analysis is not offered for exon 9.

*DMXL2:* Deletion/duplication analysis is not offered for exon 2.

*SERPINA1:* Deletion/duplication analysis is not offered for exon 3.

*DDX3X:* Sequencing analysis is not offered for exon 3.

*GBA:* c.84dupG (p.Leu 29Alafs*18), c.115+1G>A (Splice donor), c.222_224delTAC (p.Thr75del), c.475C>T (p.Arg159Trp), c.595_596delCT (p.Leu 199Aspfs*62), c.680A>G (p.Asn227Ser), c.721G>A (p.Gly241 Arg), c.754T>A (p.Phe252lle), c. 1226A>G (p.Asn409Ser), c.1246G>A (p.Gly416Ser), c.1263_1317del (p.Leu422Profs *4), c. 1297G>T (p.Val433 Leu), c.1342G>C (p.Asp448His), c.1343A>T (p.Asp448Val), c.1448T>C (p.Leu483 Pro), c. 1504C>T (p.Arg502Cys), c.1505G>A (p.Arg502His), c.1603C>T (p.Arg535Cys), c.1604G>A (p.Arg535His) variants only. Rarely, sensitivity to detect these variants may be reduced. When sensitivity is reduced, zygosity may be reported as "unknown".

*MACF1:* Sequencing analysis for exons 39 includes only cds +/- 10 bp.

*ATP8B1:* Sequencing analysis for exons 19 includes only cds +/- 10 bp.

*FAH:* Deletion/duplication analysis is not offered for exon 14.

*PGM1:* Deletion/duplication analysis is not offered for exon 11.

*EIF2AK1:* Sequencing

analysis for exons 9 includes only cds +/- 0 bp.

*PHAX* Deletion (Entire coding sequence) only.

*MFF*: Deletion/duplication analysis is not offered for exon 3.

*KCNC3:* Sequencing analysis is not offered for exon 4.

*PKD1L1:* Sequencing analysis for exons 3 includes only cds +/- 0 bp.

*AUTS2:* Sequencing analysis for exons 3 includes only cds +/- 10 bp.

*NALCN:* Sequencing analysis for exons 19 includes only cds +/-0 bp.

*ENPP1:* Sequencing analysis for exons 2 includes only cds +/- 5 bp.

*KMT2E:* Sequencing analysis for exons 6 includes only cds +/- 10 bp.

*TM4SF20*: Deletion/duplication analysis is not offered for exon 1.

*RTTN*: Sequencing analysis for exons 32 includes only cds +/- 0 bp.

*MSTO1:* Deletion/duplication analysis is not offered for exons 1-7, 10, 12-14 and sequencing analysis is not offered for exons 1-7, 10, 13-14.

*LHB:* Deletion/duplication analysis is not offered for exon 2.

*ZSWIM6:* Sequencing analysis is not offered for exon 1.

*SIK1*: Deletion/duplication analysis is not offered for exons 13-14.

*NSUN2*: Deletion/duplication analysis is not offered for exon 9.

*BRWD3*: Sequencing analysis for exons 9 includes only cds +/- 0 bp.

*TAF2:* Sequencing analysis is not offered for exon 2.

*KMT2C*: Deletion/duplication and

sequencing analysis is not offered for exons 7-8, 14-16, 18-19, 21, 24.

*SDHA*: Deletion/duplication analysis is not offered for this gene and sequencing analysis is not offered for exon 14. Sequencing analysis for exons 6-8 includes only cds +/- 10 bp.

*TSC1:* Sequencing analysis for exons 21 includes only cds +/- 10 bp.

*KDM6A*: Sequencing analysis for exons 18 includes only cds +/- 10 bp.

*PEX1*: Sequencing analysis for exons 16 includes only cds +/-0 bp.

*SAR1B:* Deletion/duplication analysis is not offered for exon 5.

*GRM1:* Sequencing analysis for exons 2 includes only cds +/-0 bp.

*GHR:* Deletion/duplication and sequencing analysis is not offered for exon 3.

*MID1:* Sequencing analysis for exons 3 includes only cds +/- 0 bp.

*GH1:* Deletion/duplication and sequencing analysis is not offered for exon 1.
