# Supplementary material for: Genetic determinants of global developmental delay and intellectual disability in Ukrainian children
Source: J Neurodev Disord. 2024 Mar 27;16:13. doi: 10.1186/s11689-024-09528-x (PMC10967201; doi:10.1186/s11689-024-09528-x)
Supplement: Supplementary file 4 — Supplementary Material 4 [file 11689_2024_9528_MOESM4_ESM.docx]

October 23, 2023

**To: Stephan Sanders, Ph.D., M.D.**

Editor-in-Chief, Journal of Neurodevelopmental Disorders

University of California, San Francisco, USA

**Subject:** Resubmission of Manuscript - JNDD-D-23-00002

Dear Dr. Sanders

Thank you for considering our titled "*Genetic determinants of global developmental delay and intellectual disability in Ukrainian children*" to the *Journal of Neurodevelopmental Disorders*” and providing us with invaluable feedback from the reviewers. We have thoroughly reviewed the comments and have made corresponding modifications to our manuscript.

Included below is a detailed account of our responses to the comments. We believe that the revisions have addressed the reviewers' concerns and have improved the quality and clarity of our manuscript. We hope that our revised manuscript will be deemed suitable for publication in the *Journal of Neurodevelopmental Disorders.*

Thank you for your time and consideration.

Sincerely yours

**Taras K. Oleksyk, Ph.D.**

Associate Professor of Biology

Oakland University

Rochester MI 48306

**Answers to Reviewer’s Comments**

**Reviewer #1:**

**Concern regarding language and structure:** *This work is of interest for GDD/IDD studies and provides important details on genes and related phenotypes as well as the possible specificities of the Ukrainian population for which this is the first large-scale study.*

*The authors have tried to address most of the comments, however, the text could still benefit from proofreading and major revisions in the context of language*

*Some parts (eg the abstract and most of the results) are difficult to follow, and some sentences are rather long (eg lines 153 to 157).*

**Answer:** We have made considerable efforts to rewrite sections that were identified as ambiguous or hard to follow, specifically the lines mentioned by the reviewer (e.g., lines 153 to 157).

**Clarity in methods section:** *I do have some notes ( some of which may be due to difficlties in comprehending the text):*

*In the methods section the retrospective nature of the study should be made more clear*

**Answer:** We have restructured the sentence in line 121 to elucidate the retrospective nature of our study as suggested.

***Table 1 formatting****: Please reformat table 1 to add the word years for age and have N (%) and other numbers in line with the text.*

**Answer:** We have reformatted Table 1 to improve its uniformity and added "years" for age and ensured the alignment of N (%) and other numbers with the text.

***Mathematical clarity:*** *Please check the numbers and maths throughout the text (e.g., in lines 191-193 the sum is 56. I suppose that the other ten are the CNVs but this is not easily comprehended).*

**Answer:** We've amended the sentence between lines 194-196 to clarify the calculations mentioned.

***Terminology correction****: VUS resolution in the family is better described as segregation analysis.*

**Answer:** We have updated the term "VUS resolution" in line 224 to "segregation analysis".

***Gene name correction in Table 2:*** *In table 2 in the second row, I think the gene is C19ORF12 and not C19OFR12.*

**Answer:** The gene name has been corrected as indicated.

***Uniform reference to variants in Table 2****: In table 2, for reason of uniformity, please refer to all variants the same way (cDNA and if possible impact on protein or other expected impact).*

**Answer:** We've standardized the representation of all variants in the table for consistency.

***Details on variants/CNVs pathogenicity:*** *I would also propose to add further details on variants/CNVs pathogenicity and characterization according to ACMG (PVS1), (PS1-4), (PM1-6), (PP1-5) (Richards et al., 2015) and Riggs et al.*

**Answer:** A column has been added to the table detailing the ACMG classification based on the criteria mentioned.

***Evaluation of autosomal dominant disorders:*** *For autosomal dominant disorders, variants are present only in patients (de novo) unless incomplete penetrance is recorded for a specific gene/disease. How were variants (especially missense ones) evaluated in the absence of segregation analysis?*

**Answer:** For diagnosed patients, the identified variants were known pathogenic variants for AD diseases. Confirmations were limited due to operational and financial constraints.

***Evaluation for autosomal recessive diseases:*** *For autosomal recessive diseases, biallelic trans configuration is required. Was that established and if so, how?*

**Answer:** We confirmed compound heterozygosity in 4 diagnosed cases using segregation analysis.

***Verification for imprinted disorders****: For imprinted disorders like Angelman syndrome, differentiation of the lesions location on the maternal or paternal allele is important. Was the deletion verified and evaluated with MS MLPA or segregation analysis of STRS within the region?*

**Answer:** Maternal deletion was confirmed by FISH in the clinic.

***Algorithm for CNV calling:*** *In respect to CNVs please refer to the algorithm applied for CNV calling. You do say in your response to the editor that it uses Invitae, maybe you could provide some more details.*

**Answer:** The computational approach for identifying Copy Number Variants (CNVs) in Next-Generation Sequencing (NGS) data obtained from targeted gene panels in Invitae relies on assessing the coverage depth of the captured regions. It computes a copy number ratio score for each region by comparing the sample's mean coverage to the mean coverage of that same region in a group of other samples (pool). Later they are confirmed. We have expanded on the computational approach for CNV identification in lines 156-158.

***Highest yield when applying WES****: In respect to the highest yield when applying WES, I would propose to avoid attributing to chance (however it is the reviewer's 2 right to call). WES does have a greater detection rate due to all the reasons described in your response to reviewer 2. It also allows characterization of novel genes linked to specific phenotypes. In addition, one could think that it might be due to the small number of patients and a possible bias in selecting them. Could it be that they had undergone extensive genetic testing ruling other defects out?*

**Answer:** We took the reviewer's feedback into account and modified our wording in line 255 to better explain the increased detection rate of WES.

**Reviewer #2:**

*The authors have responded to my comments. The language and thoroughness of the presented data are vastly improved.*

***One minor comment*** *- Table 2, last column. 'Disorder' would be a better header than 'Disease'.*

**Answer:** The header in Table 2's last column has been changed from "Disease" to "Disorder" as per the reviewer's suggestion.
